# IL-34 Expression Is Reduced in Hashimoto's Thyroiditis and Associated With Thyrocyte Apoptosis

**DOI:** 10.3389/fendo.2018.00629

**Published:** 2018-10-23

**Authors:** Shuo Wang, Yongping Liu, Na Zhao, Xuejiao Cui, Mingshi Huang, Yushu Li, Zhongyan Shan, Weiping Teng

**Affiliations:** Liaoning Provincial Key Laboratory of Endocrine Diseases, Department of Endocrinology and Metabolism, Institute of Endocrinology, The First Affiliated Hospital of China Medical University, Shenyang, China

**Keywords:** Hashimoto's thyroiditis, IL-34, thyrocyte, apoptosis, NOD.H-2^h4^ mice

## Abstract

Hashimoto's thyroiditis (HT) is a common autoimmune disease accompanied by lymphocyte infiltration and thyroid tissue destruction. IL-34 was first described in 2008, and its involvement in the development of many autoimmune diseases has been recently identified. However, whether IL-34 is a regulatory factor in HT is unclear. Here, we demonstrate that IL-34 is expressed on thyroid follicular epithelial cells and that IL-34 expression is significantly reduced in thyroid tissue in patients with HT and spontaneous autoimmune thyroiditis (SAT) models. Serum IL-34 levels in patients with HT are also significantly reduced. In addition, IL-34 is associated with thyroid autoantibodies in both thyroid tissue and serum. Furthermore, our data show that IL-34 participates in the apoptosis resistance of thyrocytes in HT induced by CSF-1R and may be a potential indicator for evaluating thyrocyte damage.

## Introduction

Hashimoto's thyroiditis (HT) is an organ-specific autoimmune disease accompanied by lymphocytic infiltration, elevated concentrations of thyroperoxidase antibody (TPOAb) and thyroglobulin antibody (TgAb), destruction of thyroid follicles (1, 2), and high TPOAb concentrations, which are significantly associated with hypothyroidism (3). These changes may be related to the destruction of thyrocytes caused by TPOAb (4). Apoptosis is a factor involved in the development of HT. Under physiological conditions, apoptosis is an important factor for maintaining homeostasis. However, an increase in apoptosis was observed in the thyroid tissue of patients with HT. During the development of HT, changes occur in the expression of apoptosis-related factors, such as Fas/FasL, caspases, and B cell lymphoma-2 (Bcl-2) (5).

IL-34 was discovered as a ligand for colony-stimulating factor-1 receptor (CSF-1R). Thus, the function of IL-34 is similar to that of macrophage colony-stimulating factor (M-CSF), which includes stimulating monocyte survival, macrophage proliferation (6), and osteoclast formation/differentiation (7). According to current research, IL-34 is observed in various organs and tissues, such as immune organs (spleen and thymus), small intestine, keratinocytes, and hair follicles. Thus far, no studies have suggested that IL-34 is expressed in thyroid tissue or clarified whether IL-34 is involved in the development of HT.

In this study, we observed a decrease in IL-34 expression in patients with HT and a negative correlation with thyroid autoantibodies. In addition, IL-34 reduced thyrocyte apoptosis through the IL-34/CSF-1R/STAT3 pathway. IL-34 may also be a potential marker of thyrocyte injury in HT.

## Methods and materials

### Mice

NOD.H-2^h4^ mice were purchased from the Jackson Laboratory (Bar Harbor, ME, USA.). Mice were bred and raised under specific pathogen-free conditions in the animal facility at China Medical University. NOD.H-2^h4^ mice at 4 weeks of age with a mean weight of 20 g were randomly divided into the control and spontaneous autoimmune thyroiditis (SAT) groups. Mice in the control group (16 mice, 8 per time-point) were given sterile water. SAT group (16 mice, 8 per time-point) were given 0.005% mg/L NaI in their drinking water for 8 or 16 weeks. Each group of mice was killed at 8 and 16 weeks after grouping. All of the animal study procedures were in accordance with the National Institutes of Health Guide for the Care and Use of Laboratory Animals and were performed according to the institutional ethical guidelines. Thyroid tissues were used for HE staining (32 mice, 8 per group), RT-PCR (32 mice, 8 mice per group) and Western blotting (32 mice, 8 per group).

### Subjects

This study was carried out in accordance with the recommendations of “the regional Ethics Committee guidelines and institutional policies, the Ethics Committee of China Medical University.” The protocol was approved by “The Ethics Committee of China Medical University (the protocol number was [2014]94).” All subjects gave written informed consent in accordance with the Declaration of Helsinki.

Thyroid tissues were obtained from 23 patients with HT who were undergoing thyroidectomies for treatment. Non-HT thyroid tissues collected from 24 paracancerous tissues from patients without HT as controls. Freshly harvested thyroid tissues were used for immunohistochemical staining (12 patients, 6 per group), RT-PCR (47 patients, 24 patients with HT and 23 patients without HT), and Western blotting (16 patients, 8 per group).

Serum was obtained from 35 patients with HT and 21 healthy controls. Patients included in the HT group were positive for at least one autoantibody. Serum samples were stored at −80°C for subsequent analysis.

### Cells and treatments

Thyroid tissues were obtained from six patients with HT for primary thyrocyte culture, and all of these subjects were euthyroid. The tissue was processed as previously described (8). Fresh thyroid tissue was scissors into small pieces of about 1 mm^3^ and incubated with Collagenase IV (1.25 mg/ml; Sigma, C1889) in Hank's solution at 37°C for 4 h. Cells suspension was filtered through a nylon cell strainer with 70 μm. The filtrate was centrifuged at 1,000 × g for 5 min. Cells were resuspended in RPMI-1640 containing 10% fetal bovine serum (FBS). Then, thyroid cells were plated in 6-well plates (1 × 10^6^ cells per well). After 24 h the supernatant containing no adherent cells was removed.

Primary thyrocytes and Nthy-ori 3-1 (a human thyroid follicular epithelial cell line from the Health Protection Agency Culture Collections, UK) were cultured in RPMI-1640, 10% FBS and Pen/Strep in 5% CO_2_ at 37 °C. Different doses of rhIL-34 (from 0 to 50 ng/mL; R&D Systems, Minneapolis, MN, USA) were used at indicated times as stimulators of thyrocytes. Primary thyrocytes were treated with rh-IL-34 (50 ng/mL) alone or plus CSF-1R inhibitor (25 μM; Tocris Cookson, Bristol, UK) for 24 h. Nthy-ori 3-1 were treated with increasing doses of the IFN-γ (from 0 to 1000 IU/mL; R&D Systems, Minneapolis, MN, USA) for 24 h.

### Western blotting

Western blotting was performed as previously described (8). Anti-IL-34 and STAT3 antibodies (1:1,000; Abcam, Cambridge, MA, UK), anti-CSF-1R and p-STAT3 antibodies (1:1,000; Cell Signaling), anti-Bax and Bcl-2 antibodies (1:1,000; BioLegend, San Diego, CA, USA), and anti-GAPDH antibodies (1:1,000; Santa Cruz, Dallas, TX, USA) were used in our study.

### Immunohistochemistry

Immunohistochemistry (IHC) was performed as previously described (8). Rabbit anti-human antibody IL-34 antibodies (1:500; Biorbyt LLC, San Francisco, CA, USA) were used at 1:500 dilutions to observe the expression of IL-34 in thyroid tissues. The staining intensity was measured by the Image-Pro Plus 5.0 software (Media Cybernetics, Inc. Silver Spring, MD USA). The mean densitometry of the image is designated as the levels of IL-34 expression.

### Measurement of serum IL-34 levels

Serum IL-34 levels were measured using an ELISA kit according to the manufacturer's directions (BioLegend, San Diego, CA, USA). Each sample was measured three times.

### RNA isolation and RT-PCR analysis

We used RT-PCR to detect mRNA expression, as previously described (8).

The following primers were used: human IL-34, F: 5′-TTGACGCAGAATGAGGAGTG-3′; R: 5′-CCCTCGTAAGGCACACTGAT-3′; Mouse IL-34, F: 5′- ATCTACAGGAGGTTCAGACATTGC-3′; R: 5′-AACAGTACAGCAGTTCCATGACC-3′; Human Bcl-2, F: 5′-ACATCCATTATAAGCTGTCGCAGAGG-3′; R: 5′- TGCAGCGGCGAGGTCCTG-3′; Human Bax: F: 5′-GATGCGTCCACCAAGAAGCTGAG-3′; R: 5′-CACGGCGGCAATCATCCTCTG-3′. Human GAPDH: F: 5′-CAGGAGGCATTGCTGATGAT-3′; R: 5′-GAAGGCTGGGGCTCATTT-3′;

Mouse GAPDH: F: 5′-GGTTGTCTCCTGCGACTTCA-3′; R: 5′- TGGTCCAGGGTTTCTTACTCC-3′. The results are shown as the mean ratio of the test molecule/GAPDH±SD.

### Flow cytometry detection of apoptosis

Apoptotic thyrocytes were detected using an Annexin V FITC Apoptosis Detection Kit (Dojindo Molecular Technologies, Rockville, MD) according to the manufacturer's directions. Thyrocyte fluorescence was analyzed by a FACS Calibur (BD Biosciences, USA) cytometer with CellQuest software.

### Statistical analysis

All data are presented as the mean ± *SD* or median. The nonparametric Mann-Whitney test and *t*-test were performed to compare the variables between groups. Associations were analyzed by using the Spearman correlation test. All graphs were performed using GraphPad Prism software version 5.0 (GraphPad Software, Inc., La Jolla, CA, USA). All western blot analysis be scanned and the abundance assessed quantitatively using Image J (NIH Image, Bethesda, MD, USA). All quantitative data analyses were performed using SPSS version 20.0 (IBM, Armonk, New York, USA), and *P* < 0.05 was considered significant.

## Results

### Reduced expression of IL-34 in the thyroid tissue of SAT mice

**Figure 1:**
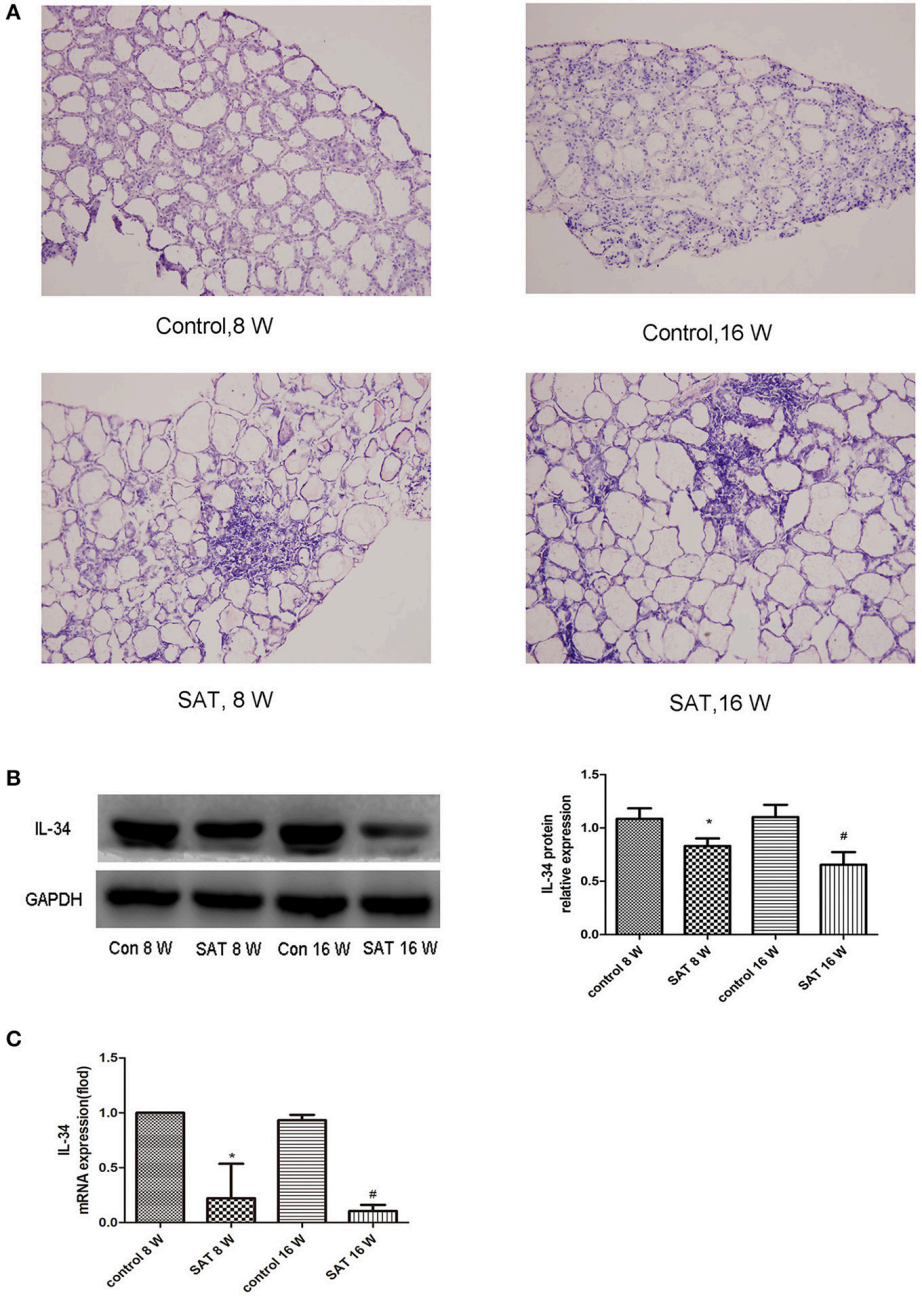
Representative histographs of HE-stained thyroid frozen sections (×200) and IL-34 expression in thyroid tissues from different groups of NOD.H-2^h4^ mice. **(A)** Large amount of lymphocyte infiltration in the SAT groups compared with that in the control groups. **(B)** IL-34 protein expression in thyroid tissues from different groups and a representative result is shown. **(C)** IL-34 mRNA expression in thyroid tissues from different groups. The results are shown as the mean ± SD. *p < 0.05 vs. control 8-week group; ^#^*p* < 0.05 vs. control 16-week group.

Thyroid follicles in the control group had a uniform morphology and moderate size. In the SAT groups, thyroid follicles were significantly enlarged, exhibited different morphologies, and contained a large amount of lymphocyte infiltration. We further examined the levels of the IL-34 in control group and SAT group by western blotting and RT-PCR. IL-34 expression was significantly lower in the SAT groups than in the control groups, but there was no difference between the groups treated with NaI water for 16 and 8 weeks (Figure 1).

### IL-34 expression in human thyroid tissue

**Figure 2:**
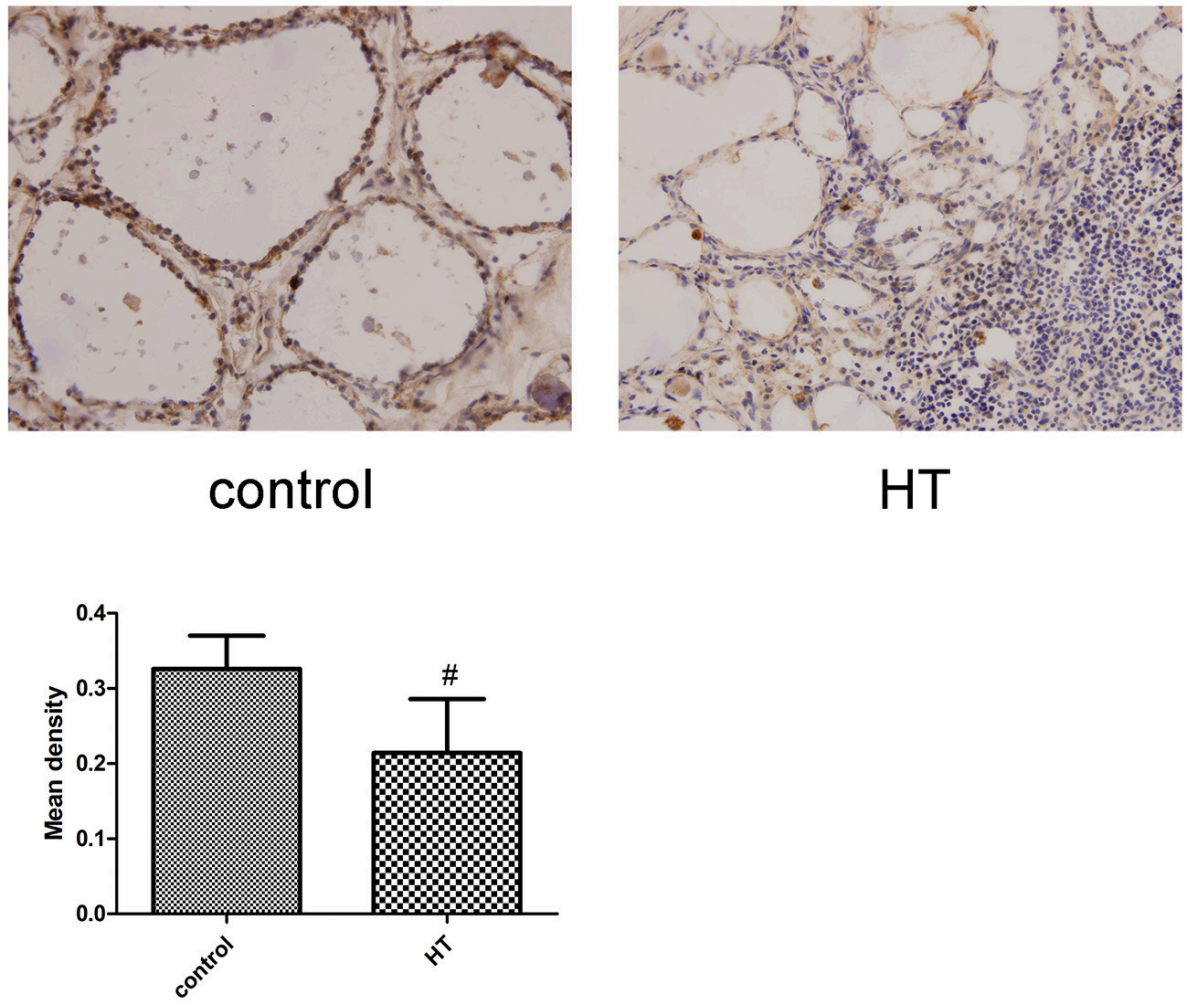
Representative staining of IL-34. IL-34 expression was analyzed by IHC staining of frozen thyroid sections from the non-HT (control) and HT groups. Each photo is representative of at least 6 thyroids examined for each group. (×400). Comparing the mean density of the non-HT and HT groups. ^#^*p* < 0.05 vs. control.

IL-34 is expressed in human thyroid follicular epithelial cells. We performed IHC analysis and measured the expression of IL-34 in human thyroid tissue. The result suggests a lower level of IL-34 expression in patients with HT compared to in patients without HT. We detected IL-34 expression in follicular epithelial cells in patients without HT. In contrast, IL-34 expressed in thyroid follicular epithelial cells and infiltrating lymphocytes in the thyroid tissue of patients with HT (Figure 2).

### Reduced IL-34 expression in the thyroid tissue of patients with hashimoto's thyroiditis and association with TgAb and TPOAb

**Figure 3:**
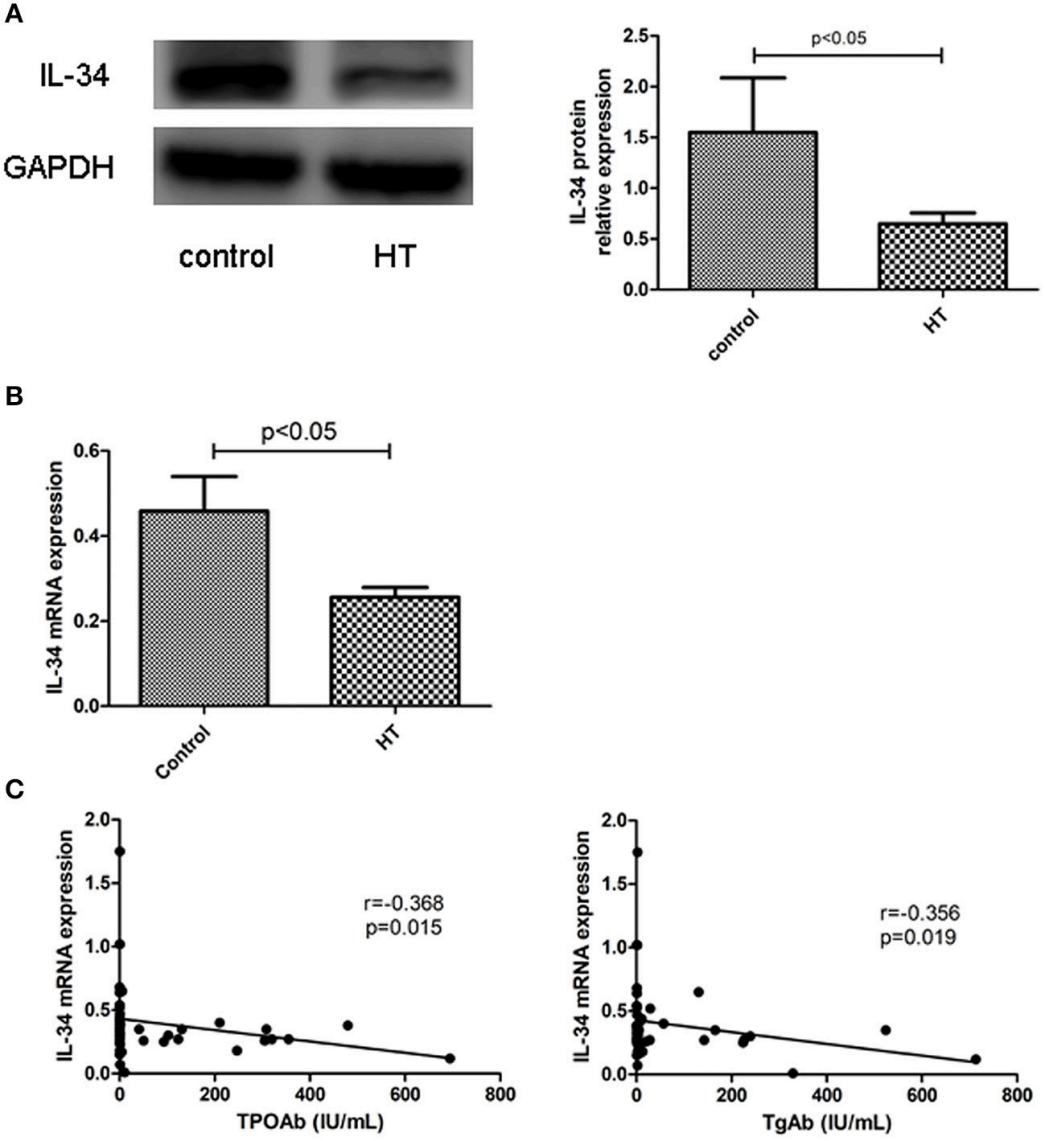
Reduced expression of IL-34 in the thyroid tissue of HT. **(A)** IL-34 protein expression in the thyroid tissue of the non-HT and HT groups and a representative result is shown. **(B)** IL-34 mRNA expression in the thyroid tissue of the non-HT (*n* = 24) and HT groups (*n* = 23). **(C)** Correlation of IL-34 mRNA expression with TgAb and TPOAb levels. The results are shown as the mean ± SD.

The clinical features of patients with HT (*n* = 24) and without HT (*n* = 23) are shown in Table 1. The level of autoantibodies in the HT group was significantly higher than that in the non-HT group (*p* < 0.001). There was no significant difference in the levels of TSH, FT4, and FT3 between the HT and non-HT groups. Then, we analyzed the correlation between the mRNA expression of IL-34 in thyroid tissue and thyroid function parameters. IL-34 protein expression in the thyroid tissue of the HT group was significantly lower than that of the non-HT group (Figure 3A), Similarly, IL-34 mRNA expression in the thyroid tissue of the HT group was significantly lower than that of the non-HT group (Figure 3B) and a negative correlation was observed between the mRNA expression of IL-34 and both TPOAb (r = −0.368, *p* = 0.015) and TgAb (r = −0.356, *p* = 0.019) (Figure 3C). IL-34 mRNA expression in thyroid tissue has no correlation with TSH (*p* = 0.151), FT3 (*p* = 0.630), and FT4 (*p* = 0.409).

**Table 1:** Comparison of baseline data between the HT and non-HT groups.

	**Control (*n* = 59)**	**HT (*n* = 44)**	***P***
Age (years)	46.01 ± 12.26	46.02 ± 11.29	0.479
Gender (F/M)	47/12	40/4	0.170
BMI (kg/m^2^)	23.47 ± 1.17	24.03 ± 3.21	0.761
TSH (mU/mL)	1.59 (1.10–2.39)	1.71 (1.07–3.91)	0.326
FT3 (pmol/L)	4.59 ± 0.55	4.36 ± 0.62	0.647
FT4 (pmol/L)	14.74 ± 2.19	14.13 ± 2.38	0.846
TPOAb (IU/mL)	0.40 (0.21–0.65)	102.63 (5.53–304.96)	< 0.001
TgAb (IU/mL)	1.33 (1.10–2.27)	29.01 (9.76–225.91)	< 0.001

*HT, Hashimoto's thyroiditis*.

### Reduced serum IL-34 levels in patients with hashimoto's thyroiditis and association with TgAb

**Figure 4:**
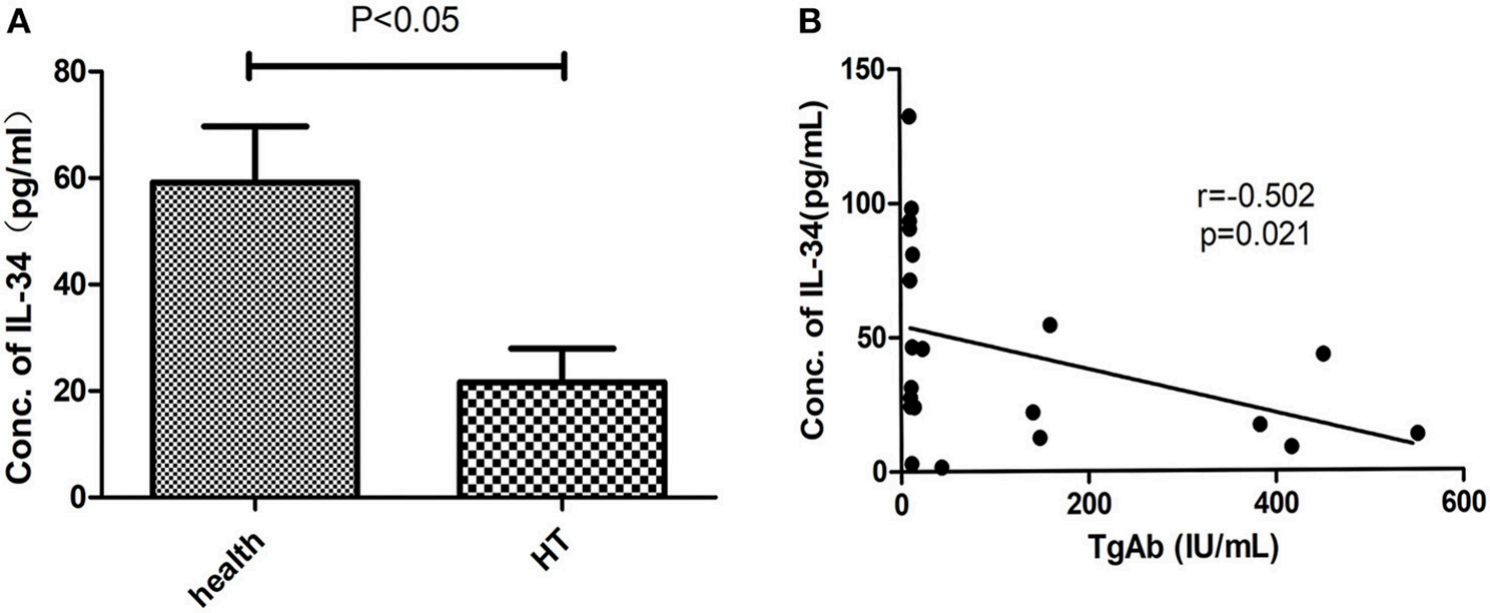
Reduced serum IL-34 levels in HT. **(A)** Serum IL-34 in patients with HT (*n* = 21) and healthy controls (*n* = 35). **(B)** The correlation between serum IL-34 levels and TgAb was calculated using Spearman's correlation. The results are shown as the mean ± SD.

The clinical features of patients with HT (*n* = 21) and healthy controls (*n* = 35) are shown in Table 1. The level of autoantibodies in the HT group was significantly higher than that in the healthy control group (*p* < 0.001). As before, we analyzed the correlation between serum IL-34 levels and thyroid function. Serum IL-34 levels in the HT group were significantly lower than those in the healthy control group (*p* < 0.05) (Figure 4A), and a significant negative correlation was observed between IL-34 and TgAb (r = −0.502, *p* = 0.021) (Figure 4B). Serum IL-34 levels has no correlation with TPOAb (*p* = 0.407), TSH (*p* = 0.07), FT3 (*p* = 0.268) and FT4 (*p* = 0.409).

### CSF-1R contributes to IL-34–dependent STAT3 activation

**Figure 5:**
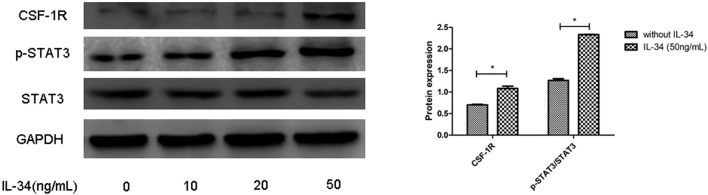
CSF1R is involved in the biological activities of IL-34. The protein expression levels of CSF1R and p-STAT3/STAT3 were evaluated by Western blotting, which was repeated three times for each experiment. The results are shown as the mean ± SD, and a representative result is shown. *p < 0.05, compared to without IL-34 stimulation group.

**Figure 6:**
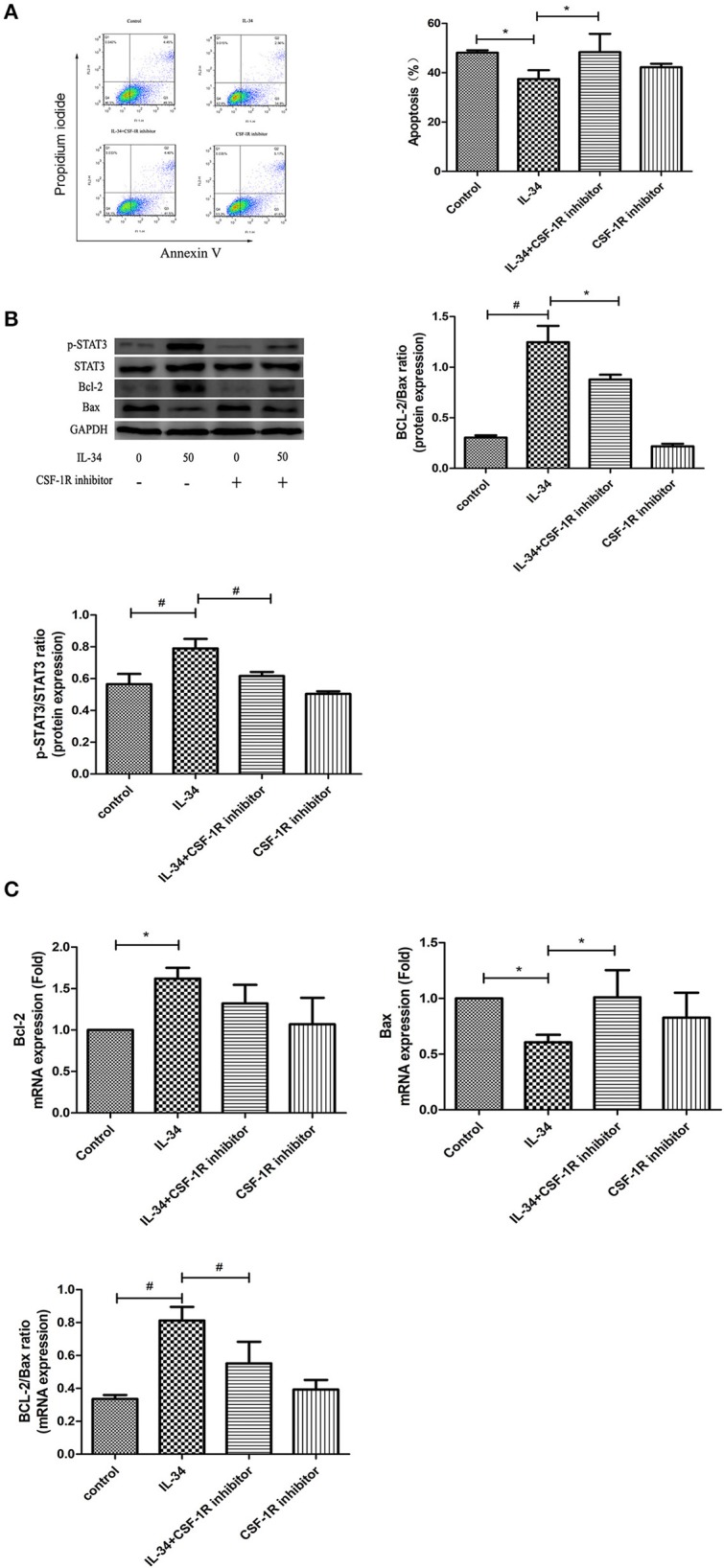
Effect of IL-34 on apoptosis of thyrocytes. Thyrocytes from patients with HT were cultured and treated with IL-34 alone or plus of CSF-1R inhibitor for 24 h. **(A)** The detection of thyrocyte apoptosis by flow cytometric analysis and statistical analysis of apoptotic cells are displayed. A representative result is shown. **(B)** The protein expression of the Bcl-2/Bax ratio was evaluated by Western blotting, and a representative result is shown. **(C)** The mRNA expression of the Bcl-2/Bax ratio was evaluated. The results are shown as the mean ± SD; *p < 0.05 and ^#^*p* < 0.01.

As a result of IL-34 treatment, increased CSF1R expression was found in thyrocytes, and p-STAT3 expression was evidently increased in a dose-dependent manner upon exposure IL-34. These findings suggested that IL-34 stimulation mediates STAT3 activation by binding to CSF1R (Figures 5, 6B).

### The IL-34/CSF-1R/STAT3 pathway is involved in the apoptosis resistance of thyrocytes in HT

As shown in Figure 6A, flow cytometric analysis demonstrated that treatment with IL-34 significantly decreased thyrocyte apoptosis compared with control (Figure 6A). Following IL-34 treatment, the protein expression of Bcl-2 increased, and the expression of Bax was decreased, resulting in an increased Bcl-2/Bax ratio (Figure 6B). A similar tendency was discovered in the mRNA expression of the Bcl-2/Bax ratio (Figure 6C). Decreased protein expression levels, including p-STAT3/STAT3and Bcl-2/Bax, were observed following treatment with CSF1R inhibitor (Figure 6B), suggesting that the IL-34/CSF-1R/STAT3 pathway is involved in the apoptosis resistance of thyrocytes in HT.

### IFN-γ-mediated decrease in IL-34 expression in Nthy-ori 3-1 cells

**Figure 7:**
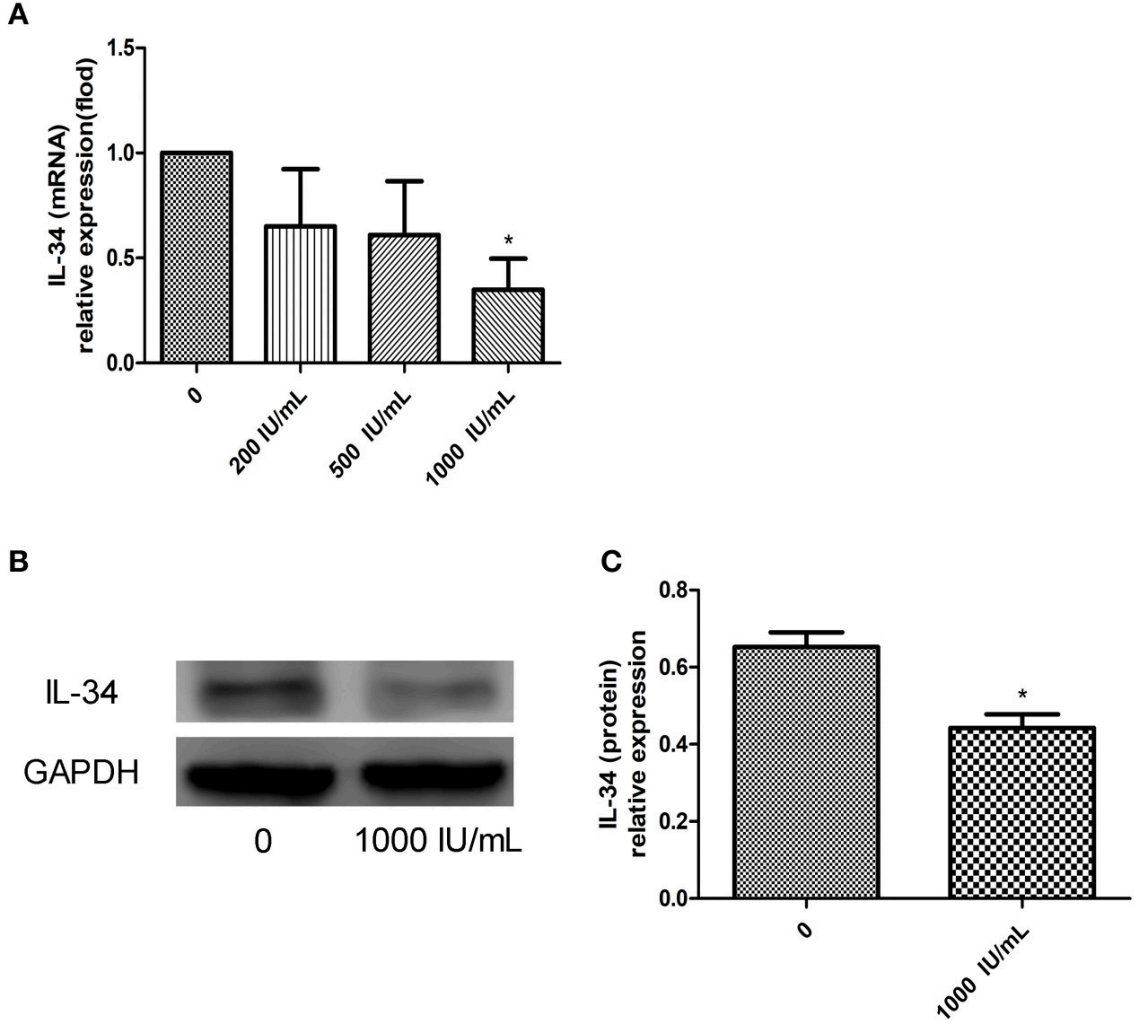
**(A–C)** Relative expression of IL-34 in Nthy-ori 3-1 cells cultured in the absence of IFN-γ or after 24 h of exposure to increasing doses of IFN-γ (from 200 to 1,000 UI/mL). The results are shown as the mean ± SD, and each experiment was repeated three times. *p < 0.05, compared to without IFN-γ stimulation group.

IL-34 expression decreased after Nthy-ori 3-1 cells were treated with IFN-γ, and IL-34 expression was significantly lower in the IFN-γ-treated group (treated with an IFN-γ concentration of 1000 UI/mL) than in the untreated group (*p* < 0.05) (Figure 7). Reduction of IL-34 expression in Nthy-ori 3-1 cells can be observed after Nthy-ori 3-1 cells damage induced by high doses of IFN-γ. We conclude that IL-34 may be associated with damage to thyrocytes.

## Discussion

First, we observed the expression of IL-34 in thyroid tissue in NOD.H-2^h4^ mice treated with NaI water for 0, 8, and 16 weeks. The expression of IL-34 in thyroid tissue was significantly lower in the SAT group than in the control groups. The expression of IL-34 was lower in the SAT 16-week group than in the SAT 8-week group, but the difference was nonsignificant. This result may have been observed because lymphocyte infiltration were no longer elevated after 12 weeks of treatment with NaI water in SAT mice.

Our study confirms for the first time that the presence of IL-34 in thyroid human tissue, and the expression of IL-34 in the thyroid tissue of patients with HT was significantly lower than that of non-HT patients. Additionally, IL-34 expression in thyroid tissue was negatively correlated with levels of TgAb and TPOAb. The serum levels of IL-34 in patients with HT were also significantly lower than those in healthy controls (~1/2 the level of the healthy control group), but the serum levels of IL-34 were negatively correlated with only the levels of TgAb. TgAb and TPOAb are important markers for the diagnosis of HT. It has been demonstrated that elevated TgAb and TPOAb indicate the disease state of HT and complement-dependent antibody-mediated cytotoxicity that can lead to thyroid tissue damage (4). Since there is a correlation between thyroid autoantibodies and IL-34, we speculated that IL-34 may also be associated with thyroid tissue damage. Thus, we stimulated Nthy-ori 3-1 cells with IFN-γ, and the effect of IFN-γ stimulation on IL-34 expression in Nthy-ori 3-1 cells was observed. IFN-γ is a vital proinflammatory factor in the pathogenesis of HT. After treatment with interferon in patients with hepatitis C, the risk of thyroiditis increases significantly. IFN-γ not only directly and indirectly destroys thyroid tissue but also promotes Th1 cell development and suppression of Th2 cell development (9). In this study, after stimulation of Nthy-ori 3-1 cells with IFN-γ, the expression of IL-34 was significantly reduced, suggesting that IL-34 may be involved in the damage of thyrocytes in HT. In allergic dermatitis, the expression of IL-34 in the skin tissue of patients with skin lesions was significantly lower than that in patients without skin lesions, and the number of Langerhans cells was also significantly reduced (10), indicating IL-34 may be an indicator of tissue damage.

Apoptosis plays an important role in HT. Increased thyroid tissue apoptosis, elevated caspase-3 expression, and decreased autophagy occur in patients with HT (11). In the process of apoptosis of thyroid tissue, the Bcl-2 family and the proapoptotic protein BH3-interacting domain death agonist (Bid) can activate caspase-8 molecules and initiate an endogenous pathway to activate apoptosis to release cytochrome C. Cytochrome C released into the cytoplasm binds to apoptosis-associated factor 1 (APAF-1) in the presence of dATP and promotes the binding of caspase-9 for the formation of apoptotic bodies. Activated caspase-9 can activate other caspases such as caspase-3 and induce apoptosis. The activation of the intrinsic pathway (mitochondria) can also directly damage the basic structure of the cell, such as DNA, and result in metabolic misalignment or the destruction of the cell cycle (12, 13). The homeostasis of thyroid tissue is very important for thyroid thyrocytes. In HT, IL-34 expression in thyroid tissue is significantly reduced. Decreased IL-34 expression in thyroid tissue may be the result of multiple factors. Therefore, we mainly investigated the decreased expression of IL-34 in thyroid tissue and its effect on thyrocytes. In response to stimulation with IL-34, the proportion of apoptotic thyroid cells was significantly reduced, the expression of proapoptosis factor Bax was decreased, the expression of antiapoptosis factor Bcl-2 was increased, and the ratio of Bcl-2/Bax was increased. Our results also proved that IL-34 can mediate the phosphorylation of STAT3 by CSF-1R, and the level of phosphorylated STAT3 increased correspondingly with the increase in the concentration of IL-34. Therefore, we hypothesize that IL-34 activates STAT3 phosphorylation via CSF-1R, thereby inhibiting the apoptosis of thyrocytes in patients with HT. IL-34 has effects on the survival and differentiation of brain microglial cells and skin Langerhans cells (14). Both CD4+ Tregs and CD8+ Tregs can produce IL-34 and participate in the immune tolerance of transplant grafts (15). In inflammatory bowel disease (IBD), IL-34 protects the integrity of the intestinal epithelium, and IL-34-differentiated macrophages show decreased IL-1β and TNF-α expression (16). In addition, CSF-1 and CSF-1R-deficient mice exhibit defects in intestinal epithelial proliferation (17). Therefore, these results suggest that IL-34 plays a protective role in IBD and can maintain the survival and proliferation of intestinal epithelial cells. Moreover, IL-34 may play an inhibitory role in acute or chronic inflammatory diseases rather than cause disease.

In conclusion, this study confirmed the presence of IL-34 in thyroid tissue. In patients with HT, IL-34 expression was significantly reduced in thyroid tissue, and IL-34 mRNA expression in thyroid tissue was negatively correlated with TgAb and TPOAb. Serum IL-34 levels in patients with HT were also significantly reduced, and serum IL-34 levels were negatively correlated with TgAb. IL-34 can reduce the apoptosis of thyrocytes in HT. In addition, high-dose INF-γ stimulation of Nthy-ori 3-1 cells can cause a significant reduction in IL-34 expression. Decreased expression of IL-34 in thyroid tissue also occurred in SAT mice. These results suggest that IL-34 could promote apoptosis resistance in thyrocytes in HT and may be a potential indicator for evaluating thyrocyte damage.

Our study has some limitations. First, IL-34 expression in thyroid tissue was negatively correlated with the levels of TgAb and TPOAb, but the serum levels of IL-34 were negatively correlated with only the levels of TgAb. At present, the mechanism of how TgAb and TPOAb work in HT is not clear. Therefore, we are unable to explain the reasons for this result. Second, *in vitro* studies do not fully represent studies *in vivo*. Nevertheless, *in vitro* studies are necessary and widely accepted to provide safety information for future *in vivo* study.

## Author contributions

SW, YoL, ZS, WT, and YuL contributed conception and design of the study. SW, NZ, and MH organized the database. SW and XC performed the statistical analysis. SW and NZ wrote the first draft of the manuscript. SW and MH wrote sections of the manuscript. All authors contributed to manuscript revision, read, and approved the submitted version.

### Conflict of interest statement

The authors declare that the research was conducted in the absence of any commercial or financial relationships that could be construed as a potential conflict of interest.
